# A Superfast Super-Resolution Method for Radar Forward-Looking Imaging

**DOI:** 10.3390/s21030817

**Published:** 2021-01-26

**Authors:** Weibo Huo, Qiping Zhang, Yin Zhang, Yongchao Zhang, Yulin Huang, Jianyu Yang

**Affiliations:** School of Information and Communication Engineering, University of Electronic Science and Technology of China, No. 2006, Xiyuan Ave, West Hi-Tech Zone, Chengdu 611731, China; hwbuyi@163.com (W.H.); yinzhang@uestc.edu.cn (Y.Z.); yongchaozhang@uestc.edu.cn (Y.Z.); yulinhuang@uestc.edu.cn (Y.H.); jyyang@uestc.edu.cn (J.Y.)

**Keywords:** super-resolution, radar imaging, Gohberg-Semencul representation, vector extrapolation

## Abstract

The super-resolution method has been widely used for improving azimuth resolution for radar forward-looking imaging. Typically, it can be achieved by solving an undifferentiable $L_{1}$ regularization problem. The split Bregman algorithm (SBA) is a great tool for solving this undifferentiable problem. However, its real-time imaging ability is limited to matrix inversion and iterations. Although previous studies have used the special structure of the coefficient matrix to reduce the computational complexity of each iteration, the real-time performance is still limited due to the need for hundreds of iterations. In this paper, a superfast SBA (SFSBA) is proposed to overcome this shortcoming. Firstly, the super-resolution problem is transmitted into an $L_{1}$ regularization problem in the framework of regularization. Then, the proposed SFSBA is used to solve the nondifferentiable $L_{1}$ regularization problem. Different from the traditional SBA, the proposed SFSBA utilizes the low displacement rank features of Toplitz matrix, along with the Gohberg-Semencul (GS) representation to realize fast inversion of the coefficient matrix, reducing the computational complexity of each iteration from $O(N^{3})$ to $O(N^{2})$. It uses a two-order vector extrapolation strategy to reduce the number of iterations. The convergence speed is increased by about 8 times. Finally, the simulation and real data processing results demonstrate that the proposed SFSBA can effectively improve the azimuth resolution of radar forward-looking imaging, and its performance is only slightly lower compared to traditional SBA. The hardware test shows that the computational efficiency of the proposed SFSBA is much higher than that of other traditional super-resolution methods, which would meet the real-time requirements in practice.

## 1. Introduction

Radar forward-looking imaging plays an important role in precision guidance, autonomous driving, surface mapping and so on. The bistatic synthetic aperture radar (bistatic SAR) is an effective technology for radar forward-looking imaging [1,2,3]. However, due to the need for bistatic cooperation, it is not applicable in some applications due to the limitation of the platform. Monopulse radar can realize forward-looking imaging [4], but the imaging scene is greatly limited according to the principle of angle measurement, and the multiple scatters within one beam and one range bin cannot be resolved. The array antenna can also obtain a high-resolution image in the forward-looking region [5], but it is usually not applicable due to platform limitations.

Recent research has been focused on achieving forward-looking imaging using a real-aperture radar. This radar can realize forward-looking imaging by antenna scanning [6,7]. However, since the azimuth resolution $\rho_{a}$ is related to antenna size, i.e.,

(1) $$\rho_{a}\propto R\frac{\lambda }{L}$$

where *R* is working distance, λ is the wave length and *L* is antenna size, the azimuth resolution is limited to antenna size. Hence, many researchers have been devoted to solving this problem using signal processing methods.

In mathematics, research shows that the azimuth echo of real-aperture radar can be modeled as the convolution of the antenna pattern and target scattering distribution [8,9,10]. Therefore, current studies are devoted to improving radar azimuth resolution by deconvolution methods, such as Wiener filtering [11], Tikhonov regularization (TREGU) [12], truncate singular value decomposition (TSVD) [13], the Richardson–Lucy (RL) method [14,15], iterative adaptive approach (IAA) [16,17] and so on. However, the improvement in resolution is limited.

Sparse regularization is an effective method for improving the azimuth resolution because the target of interest is usually sparse in radar forward-looking imaging. In the previous work, we conducted in-depth research on sparse regularization methods [18,19]. Typically, the sparse regularization method requires solving an $L_{1}$ regularization problem [20,21]. Because the $L_{1}$ norm is not differentiable, the solution is full of challenges. Split Bregman algorithm (SBA), as an efficient iterative algorithm, has been widely used to solve the challenging problem in many fields, such as imaging deblurring [22], radar super-resolution imaging [23], compressed sensing [24] and so on. In [18], SBA was utilized to improve the azimuth resolution of radar forward-looking imaging. The results show that SBA has better performance than traditional methods in resolution improvement and noise suppression. However, it is necessary to perform matrix inversion in each iteration. The problem is that the computational complexity of matrix inversion is as high as the third power of *N*, which leads to the high computational complexity of the algorithm. In radar imaging, the echo dimension is usually large, and the existence of inversion seriously restricts the calculation efficiency. In our recent study, the high computational complexity of matrix inversion has been decreased by the Gohberg-Semencul (GS) representation [19] (We named it FSBA in [19]), which reduces the computational complexity of each iteration from $O(N^{3})$ to $O(N^{2})$; however, it usually takes hundreds of iterations to converge to the optimal solution. In practical applications, we need the radar to provide clear target information in the imaging area in real time, which confers high requirements for the real-time performance of the radar. The iterations should be further reduced to meet the real-time requirement.

Aiming at the low azimuth resolution of radar forward-looking imaging and the high computational complexity of traditional SBA, a superfast SBA (SFSBA) is proposed in this paper. The low azimuth resolution is improved by solving an $L_{1}$ regularization problem. Different from traditional SBA, the proposed SFSBA firstly utilizes the Toeplitz structure of the coefficient matrix, along with the low displacement rank feature of the Toeplitz matrix and realizes fast inversion through the GS representation, reducing the computational complexity of each iteration to $O(N^{2})$. Then, the iterations are reduced by a two-order vector extrapolation strategy. After vector extrapolation, the next iteration will not start from the current iteration point, but from the predicted point extrapolated from the current iteration point. The application of vector extrapolation will greatly accelerate the convergence speed of the algorithm. Compared with FSBA in [19], not only the computational complexity of each iteration is decreased, but also the number of iterations is reduced. Finally, the superior performance of the proposed SFSBA is verified by experiments.

The reminder of the paper is structured as follows. In Section 2, super-resolution imaging is achieved by a traditional solution. In Section 3, the proposed SFSBA is deduced in detail. In Section 4, the simulation and real data processing are reported to verify the performance of the proposed algorithm. The conclusion is discussed in Section 5.

## 2. Super-Resolution with Traditional SBA

Recent research has proved that after pulse compression and range walk correction [25], the azimuth echo in radar forward-looking imaging can be modeled as a convolution of target scattering distribution and the antenna pattern [4], i.e.,

(2) y=Hσ+n

where y is the noise-polluted echo, H is the convolution matrix which is structured by the antenna pattern, σ is the target scattering, n is noise and

(3) $$H=\left[\begin{array}{cccc} h_{1} & 0 & \cdots & 0\\ h_{2} & h_{1} & \ddots & \vdots \\ \vdots & h_{2} & \ddots & 0\\ h_{L} & \vdots & \ddots & h_{1}\\ 0 & h_{L} & \vdots & h_{2}\\ \vdots & \ddots & \ddots & \vdots \\ 0 & \cdots & 0 & h_{L} \end{array}\right]$$

The target scattering σ can be estimated by solving following $L_{1}$ regularization problem, i.e.,

(4) $$\hat{\sigma }=\frac{\mu }{2}{∥H\sigma -y∥}_{2}^{2}+{∥\sigma ∥}_{1}$$

where $\hat{\sigma }$ is the estimation of σ, μ is the regularization parameter used to balance the resolution improvement and noise amplification and ${∥\sigma ∥}_{1}=\sum_{i}\left|\sigma_{i}\right|$.

To solve problem (4), the SBA is used in our work. Because the ${∥\sigma ∥}_{1}$ is not differentiable, we first employ a variable g to relax it, which leads a constraint problem, i.e.,

(5) $$\begin{array}{c} \hat{\sigma }=\frac{\mu }{2}{∥H\sigma -y∥}_{2}^{2}+{∥g∥}_{1}\\ s.t\;g=\sigma \end{array}$$

The constraint problem (5) can be converted into an unconstraint problem, i.e.,

(6) $$\hat{\sigma }=\frac{\mu }{2}{∥H\sigma -y∥}_{2}^{2}+\frac{\lambda }{2}{∥g-\sigma ∥}_{2}^{2}+{∥g∥}_{1}$$

where λ is a positive parameter.

Based on Bregman iterative criterion, an optimization problem needs to be minimized, i.e., [18]

(7) $$\left(\sigma^{k},g^{k}\right)=\min_{\sigma ,g}{∥g∥}_{1}+\frac{\mu }{2}{∥H\sigma -y∥}_{2}^{2}+\frac{\lambda }{2}{∥g^{k-1}-\sigma -b^{k-1}∥}_{2}^{2}$$

(8) $$b^{k}=b^{k-1}+\left(\sigma^{k}-g^{k}\right)$$

One of the advantages of the SBA is variable splitting, which benefit to simplify calculation. The solution to the optimization problem (7) and (8) can be achieved by solving three subproblems.

Subproblem 1: Solving σ problem. From problem (7), σ problem can be obtained by fixing g and b,

(9) $$\sigma^{k}=\min_{\sigma }\frac{\mu }{2}{∥H\sigma -y∥}_{2}^{2}+\frac{\lambda }{2}{∥g^{k-1}-\sigma -b^{k-1}∥}_{2}^{2}$$

which can be easily solved by direct derivation and using the Gauss–Seidel iteration, i.e.,

(10) $$\sigma^{k}={\left(\mu H^{T}H+\lambda I\right)}^{-1}\left(\mu H^{T}y+\lambda \left(g^{k-1}-b^{k-1}\right)\right)$$

where I is the identity matrix.

Subproblem 2: Solving g problem. From problem (7), g problem can be obtained by fixing σ and b,

(11) $$g^{k}=\min_{g}\frac{\lambda }{2}{∥g-\sigma^{k}-b^{k-1}∥}_{2}^{2}+{∥g∥}_{1}$$

which can be solved using the iterative shrinkage threshold algorithm [26], i.e.,

(12) $$g^{k}=\varsigma \left(\sigma^{k}+b^{k-1},1/\lambda \right)$$

where $\varsigma \left(x,\eta \right)=\mathrm{sign}\left(x\right)\max\left(\left|x\right|-\eta ,0\right)$.

Subproblem 3: Solving b problem. The b problem can be solved by iterative (8).

## 3. Super-Resolution with the Proposed SFSBA

Although the $L_{1}$ regularization problem (4) has been solved by traditional SBA, which is iterating (10), (12) and (8), the imaging efficiency is limited. From the solution, we can find that the main computational complexity comes from (10). In this section, the superfast accelerate strategy is proposed to reduce the computational complexity and number of iterations.

### 3.1. Fast Inversion of Toeplitz Matrix

For convenience, we rewritten (10) as

(13) $$\sigma^{k}=R^{-1}x^{k-1}$$

with $R=\mu H^{T}H+\lambda I$ and $x^{k-1}=\mu H^{T}y+\lambda \left(g^{k-1}-b^{k-1}\right)$. From the structure of matrix H and I, the matrix R is a Toeplitz matrix. As a result, the $R^{-1}$ of (18) can be effectively solved by suitable GS representations, and the computation of (18) can be implemented more efficiently by fast Toeplitz-vector multiplication methods.

The accelerated strategy first estimates the autoregressive coefficients a and prediction error e from the Yule–Walker AR equations: [27]:

(14) $$r_{1}+a_{2}r_{2}^{*}+\cdots +a_{X}r_{X}^{*}=e$$

(15) $$\left[\begin{array}{cccc} r_{1} & r_{2}^{*} & \cdots & r_{X-1}^{*}\\ r_{2} & r_{1} & \cdots & \vdots \\ \vdots & \vdots & \ddots & r_{2}^{*}\\ r_{X-1} & r_{X-2} & \cdots & r_{1} \end{array}\right]\left[\begin{array}{c} a_{2}\\ a_{3}\\ \vdots \\ a_{X} \end{array}\right]=\left[\begin{array}{c} -r_{2}\\ -r_{3}\\ \vdots \\ -r_{X} \end{array}\right]$$

Define

(16) $$w=\left[\begin{array}{c} 1\\ a \end{array}\right]\frac{1}{\sqrt{e}}\overset{\Delta }{=}{\left(\begin{array}{cccc} w_{1} & w_{2} & \cdots & w_{N} \end{array}\right)}^{T}$$

(17) $$t=\left[\begin{array}{c} 1\\ {\tilde{a}}^{*} \end{array}\right]\frac{1}{\sqrt{e}}\overset{\Delta }{=}{\left(\begin{array}{cccc} t_{1} & t_{2} & \cdots & t_{N} \end{array}\right)}^{T}$$

Utilizing the GS representation, the inversion of R can be expressed as [28,29]

(18) $$R^{-1}=WW^{H}-TT^{H}$$

with

(19) $$W=\left[\begin{array}{cccc} w_{1} & 0 & \cdots & 0\\ w_{2} & w_{1} & \ddots & \vdots \\ \vdots & \vdots & \ddots & 0\\ w_{N} & w_{N-1} & \cdots & w_{1} \end{array}\right]$$

(20) $$T=\left[\begin{array}{cccc} t_{1} & 0 & \cdots & 0\\ t_{2} & t_{1} & \ddots & \vdots \\ \vdots & \vdots & \ddots & 0\\ t_{N} & t_{N-1} & \cdots & t_{1} \end{array}\right]$$

Then, (13) can be rapidly solved using the GS representation, i.e.,

(21) $$\sigma^{k}=\left(WW^{H}-TT^{H}\right)x^{k-1}=WW^{H}x^{k-1}-TT^{H}x^{k-1}$$

Define matrix

(22) $$W_{1}=\left[\begin{array}{cccc} w_{1} & & & \\ w_{2} & w_{1} & & \\ \vdots & w_{2} & \ddots & \\ w_{N} & \vdots & \ddots & w_{1}\\ & w_{N} & & w_{2}\\ & & \ddots & \vdots \\ & & & w_{N} \end{array}\right]$$

(23) $$W_{2}=\left[\begin{array}{cccc} w_{N}^{*} & & & \\ \vdots & \ddots & & \\ w_{2}^{*} & & \ddots & \\ w_{1}^{*} & w_{2}^{*} & & w_{N}^{*}\\ & w_{1}^{*} & \ddots & \vdots \\ & & \ddots & w_{2}^{*}\\ & & & w_{1}^{*} \end{array}\right]$$

Since $W_{1}$ and $W_{2}$ have a cyclic matrix structure, the product of $W_{1}$ or $W_{2}$ with a vector can be obtained by fast Fourier transform (FFT). We can find that W can be obtained by intercepting the 1 to *N* rows of $W_{1}$, and the $W^{H}$ can be obtained by intercepting the *N* to 2N−1 rows of $W_{2}$. Therefore, the multiplication of matrix W and vector can be seen as the 1 to *N* elements of the FFT of w and the vector. The multiplication of matrix $W^{H}$ and vector can be seen as the *N* to 2N−1 elements of the FFT of $\tilde{w}$ and the vector, where $\tilde{w}={\left[\begin{array}{cccc} w_{N}^{*} & w_{N-1}^{*} & \cdots & w_{1}^{*} \end{array}\right]}^{T}$. For the same reason, $TT^{H}x^{k}$ can also be calculated by two FFT and truncations.

### 3.2. Accelerating Iteration by Vector Extrapolation

With the GS representation, the computational complexity of each iteration has been reduced to $O(N^{2})$. This subsection uses a vector extrapolation strategy to reduce the number of iterations. As an effective method to improve the convergence rate, vector extrapolation is widely used to accelerate the iterative algorithm [30,31]. This method uses the results of previous iterations to extrapolate the next iteration point.

**Figure 1:**
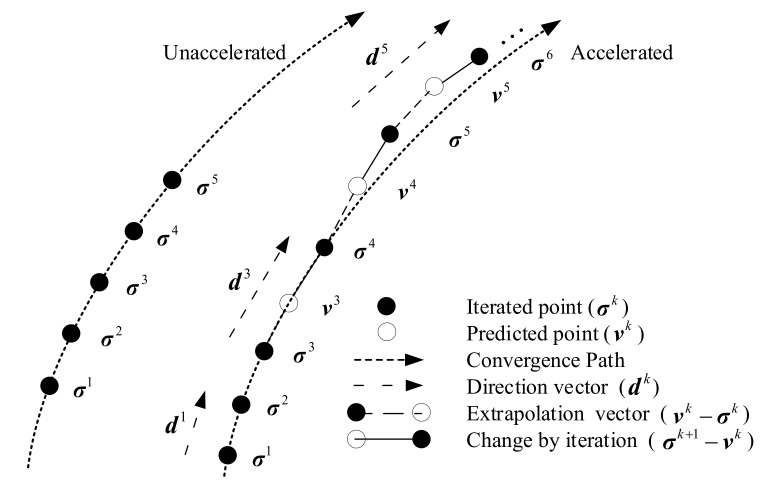
Vector extrapolation diagram.

As shown in Figure 1, $\sigma^{k}$ is the iterated point, $v^{k}$ is the predicted point extrapolated by $\sigma^{k-1}$ and $\sigma^{k-2}$, $d^{k}$ is the direction vector used to control the extrapolate direction. For each extrapolation, $d^{k}$ can be obtained by

(24) $$d^{k}=\sigma^{{}_{k}}-\sigma^{{}_{k-1}}$$

The key of vector extrapolation is to obtain the predicted point $v^{k}$. It has been demonstrated that $v^{k}$ can be obtained by Taylor expansion of $\sigma^{k}$, i.e.,

(25) $$v^{k}=\sigma^{k}+\eta_{k}\nabla \sigma^{k}+\frac{1}{2!}\eta_{k}^{2}\nabla^{2}\sigma^{k}+\cdots +\frac{1}{n!}\eta_{k}^{n}\nabla^{n}\sigma^{k}$$

where $\eta_{k}$ is the acceleration parameter, which provides a correction step to adjust the step length and guarantee the stability of the solution, $\nabla^{n}\sigma^{k}$ is the *n*-order difference at point $\sigma^{k}$. After that, (12) and (8) can be realized by iterating

(26) $$g^{k}=\varsigma \left(v^{k}+b^{k-1},1/\lambda \right)$$

(27) $$b^{k}=b^{k-1}+\left(v^{k}-g^{k}\right)$$

In fact, the higher the order of extrapolation, the more accurate the result. However, high-order extrapolation suffers from high computational complexity to obtain an accurate acceleration parameter $\eta_{k}$ [32]. Therefore, two-order vector extrapolation is utilized, and the predicting point is obtained by

(28) $$v^{k}=\sigma^{k}+\eta_{k}\nabla \sigma^{k}+\frac{1}{2!}\eta_{k}^{2}\nabla^{2}\sigma^{k}$$

where $\nabla \sigma^{k}$ is the gradient of $\sigma^{k}$, $\nabla^{2}\sigma^{k}$ is the second order gradient of $\sigma^{k}$.

Finally, the acceleration parameter is obtained [32], i.e.,

(29) $$\eta_{k}=\sqrt{\frac{{\left(d^{k-1}\right)}^{T}d^{k-1}}{{\left(d^{k-2}\right)}^{T}d^{k-2}}}\;,\;0<\eta_{k}<1$$

The proposed SFSBA is listed in Table 1.

### 3.3. Analysis of Computational Performance

The computational complexities of traditional SBA and the proposed SFSBA are as follows.

For traditional SBA, it has been indicated that the computational complexity comes from (10), and its computational complexity is $O(\left(K+1\right)N^{3}+5KN^{2}+3KN+2N\log N)$ [19].

For the proposed SFSBA, (10) is replaced by (21), and the computational complexity is decreased by the GS representation and vector extrapolation. Firstly, the autoregressive coefficients a and prediction error e are obtained by the Levinson–Durbin algorithm, and the computational complexity is $O\left({\left(N-1\right)}^{2}\right)$. Then, the solution of (21) can be achieved by four Toeplitz-vector production, and the computational complexity is O(14Nlog(2N)+NlogN+4N) [33]. Hence, the computational complexity of each iteration is $O({\left(N-1\right)}^{2}+14N\log\left(2N\right)+N\log N)$. After vector extrapolation, the iterations become κ, and $\left(\kappa \ll K\right)$. As a result, the computational complexity of the proposed SFSBA is $O(\kappa \left({\left(N-1\right)}^{2}+14N\log\left(2N\right)\right)+N\log N)$.

**Figure 2:**
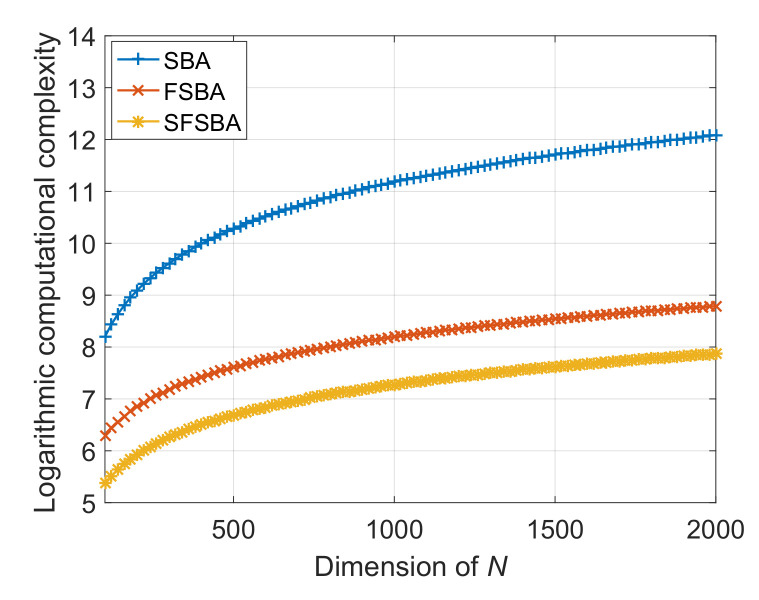
Logarithmic computational complexity of SBA, FSBA and SFSBA.

In order to intuitively compare the computational complexity of the proposed method with that of traditional SBA and FSBA, we empirically let K=150, κ=18, and plot the logarithmic computational complexity curves, as shown in Figure 2. It shows that the computational complexity of the proposed SFSBA is much lower than that of traditional SBA and FSBA, illustrating that the proposed SFSBA has a greater computational efficiency advantage compared to SBA and FSBA.

## 4. Performance Validation

In this section, we first perform a simulation and process real data to verify the performance of the proposed SFSBA in resolution improvement. The parameter of the proposed SFSBA is determined by *L*-curve method [19]. Then, the improvement in computing efficiency is demonstrated by hardware testing. As a reference, the experimental results are compared with some traditional methods, including TSVD, TREGU, RL, IAA, traditional SBA and FSBA.

### 4.1. Simulation

**Figure 3:**
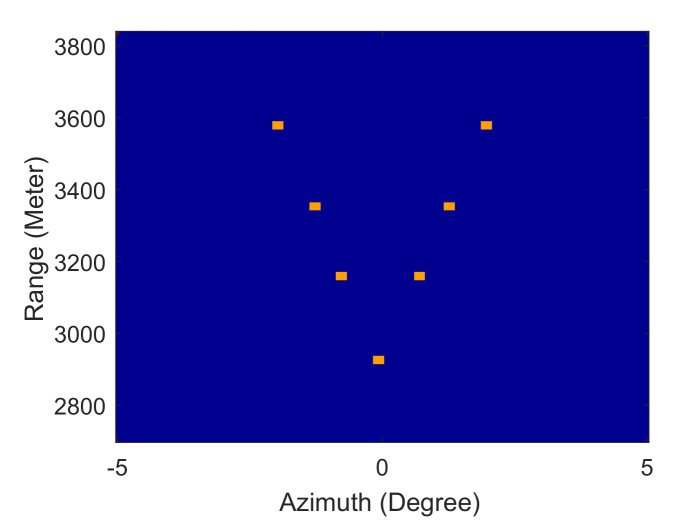
Original scene of the simulation.

For the simulation, the antenna pattern is a $sinc^{2}$ function which defined as $\mathrm{sinc}\left(x\right)=\sin\left(\pi x\right)/\left(\pi x\right)$. The beam width is 3.5${}^{\circ }$. The detailed parameters are listed in Table 2. The original scene covers fourth-lines points at different range bins, as Figure 3 shows. The intervals of adjacent targets were 3.4${}^{\circ }$, 2${}^{\circ }$ and 1.2${}^{\circ }$, respectively. Because the intervals of adjacent targets are smaller than beam width, they will not distinguished in the real beam echo. In addition, the quality of the radar image is also affected by bandwidth and pulse repetition frequency (PRF) in practice. The larger the bandwidth of the transmitted signal, the higher the signal-to-noise ratio (SNR). At a specific scanning speed, the larger the PRF is, the more the azimuth samples are. When the azimuth samples reach a certain amount, we can perform incoherent accumulation in the azimuth, which can also improve the image SNR. High SNR is beneficial to improve the super-resolution capability and stability of the algorithm.

**Figure 4:**
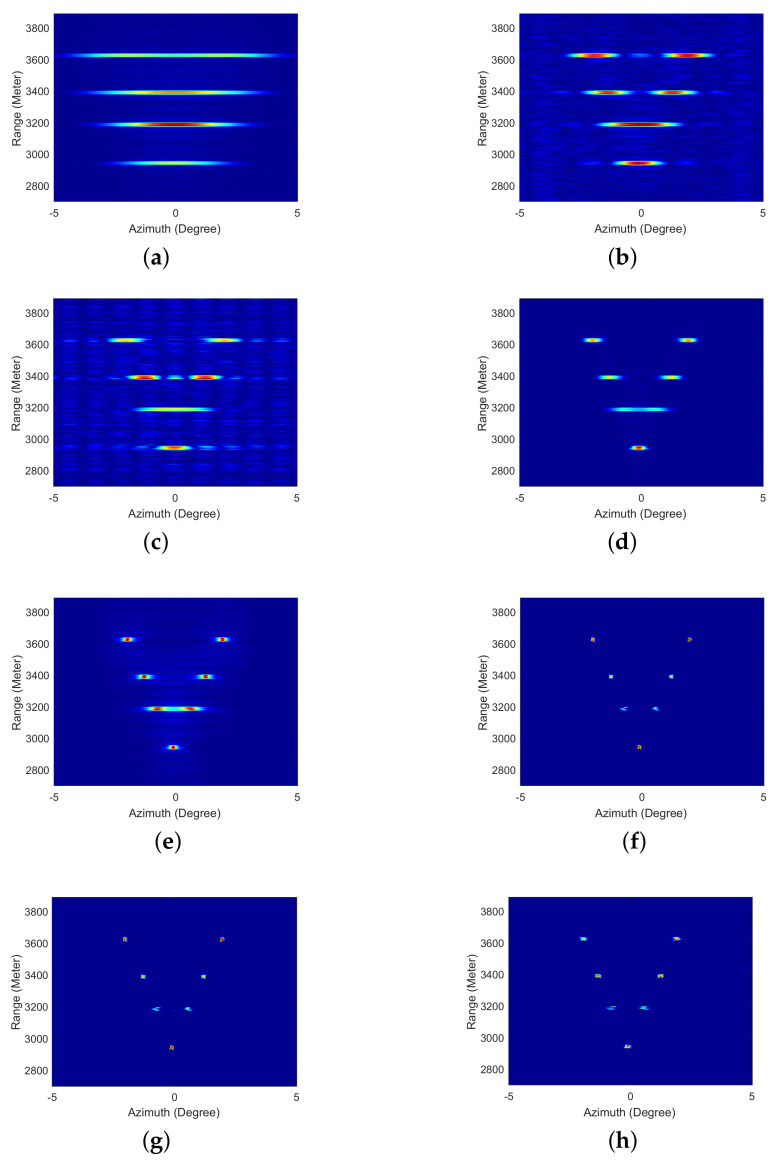
Simulation results of different methods with a signal-to-noise ratio (SNR) of 20 dB. (**a**) Real beam echo, (**b**) Result of Tikhonov regularization (TREGU), (**c**) Result of truncate singular value decomposition (TSVD), (**d**) Result of the Richardson–Lucy (RL) method, (**e**) Result of iterative adaptive approach (IAA), (**f**) Result of SBA, (**g**) Result of FSBA, (**h**) Result of SFSBA.

The simulation results are shown in Figure 4. Figure 4a is the real beam echo polluted by Gaussian noise, and the SNR is 20 dB. We can find that all the adjacent targets cannot be distinguished. Figure 4b shows that the resolution improvement of TREGU is very limited. The adjacent targets in the third line cannot be distinguished, and the noise is amplified. Figure 4c is the result of TSVD. The performance of TSVD is similar to the TREGU. From the Figure 4d,e, RL and IAA can further suppress the noise, but the third-line adjacent targets cannot be completely distinguished. SBA, FSBA and the proposed SFSBA can not only distinguish all the adjacent targets, but also supress the noise, as Figure 4f–h shows. By comparing, we can see that the result of FSBA is almost the same as the result of SBA. The proposed SFSBA also has good super-resolution performance. The adjacent targets are distinguished and the noise is suppressed.

**Figure 5:**
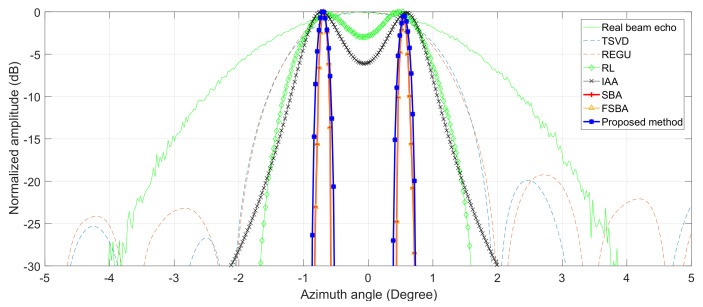
Profiles of adjacent targets.

The profiles of the third-line adjacent targets are plotted in Figure 5. It can be seen that SBA, FSBA and the proposed SFSBA can completely distinguish the adjacent targets when other methods cannot, and the profiles of SBA and FSBA completely overlap. In addition, the noise suppression ability of SBA, FSBA and the proposed SFSBA is better than other methods. By comparing the SFSBA with SBA and FSBA, it was found that although the noise suppression ability of SFSBA decreased after acceleration, the noise level was very low, lower than −25 dB, and the noise suppression ability was better than that of TREGU, TSVD, RL and IAA.

In order to quantitatively evaluate the results of different methods, we first calculate the the mean square error (MSE) to measure the similarity between the super-resolution result and the original scene. The MSE is defined as:

(30) $$MSE=\frac{1}{M_{c}}\sum_{i=1}^{M_{c}}\left(\frac{1}{MN}{∥\hat{\sigma_{i}}-\sigma ∥}_{2}^{2}\right)$$

where $M_{c}$ is the number of Monte-Carlo experiments, and $M_{c}=100$ in our simulation, $\hat{\sigma_{i}}$ is the estimation of the *i*-th Monte-Carlo experiment. Beam sharpening ratio (BSR) is employed to measure the resolve ability. BSR is defined as the ratio of beam width before and after super-resolution of a single target. In addition, image entropy of different processing results are used to measure the clarity of image. Image entropy is defined as follows:

(31) $$E=-\sum_{i=0}^{1}p_{i}\log_{2}p_{i}$$

where *E* is the entropy, $p_{i}$ is the proportion of pixels whose gray value is *i* after normalization. According to the principle of minimum entropy, smaller entropy results in a clearer image [34]. All the results are shown in Table 3. From the table, it can be seen that the performance of SBA, FSBA and the proposed SFSBA are better than that of other methods. After acceleration, the performance of the proposed SFSBA decreases by a certain degree, but it is still much better than that of the TREGU, TSVD, RL and IAA methods. To achieve superior performance, the iterations of SBA and FSBA was 200, but the proposed SFSBA was only 25. After vector extrapolation, the convergence speed of the algorithm was increased by 8 times.

### 4.2. Real Data Verification

In this section, two real data are processed by different algorithms to demonstrate the super-resolution performance in practice.

#### 4.2.1. Real Data of Ginkgo Avenue

**Figure 6:**
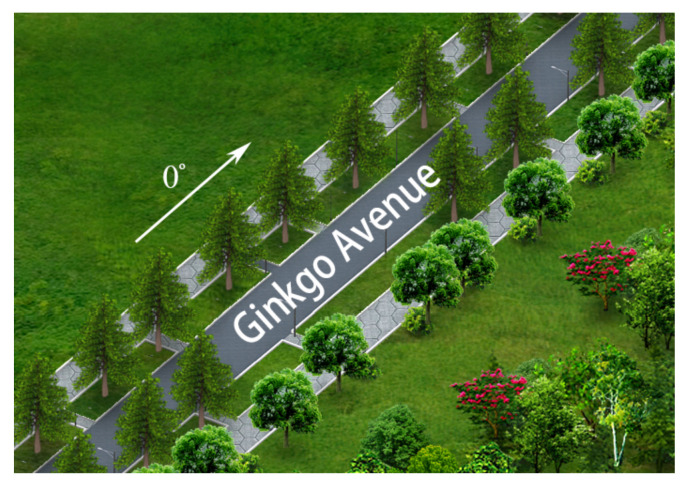
Optical scene of Ginkgo Avenue data.

The first real data were collected on Ginkgo Avenue, University of Electronic Science and technology of China, Chengdu, China. The system parameters are listed in Table 4. The optical scene of the Ginkgo Avenue is shown in Figure 6, where the arrow refers to the zero degree direction. In this experiment, the trees on both sides of the avenue are our focus.

**Figure 7:**
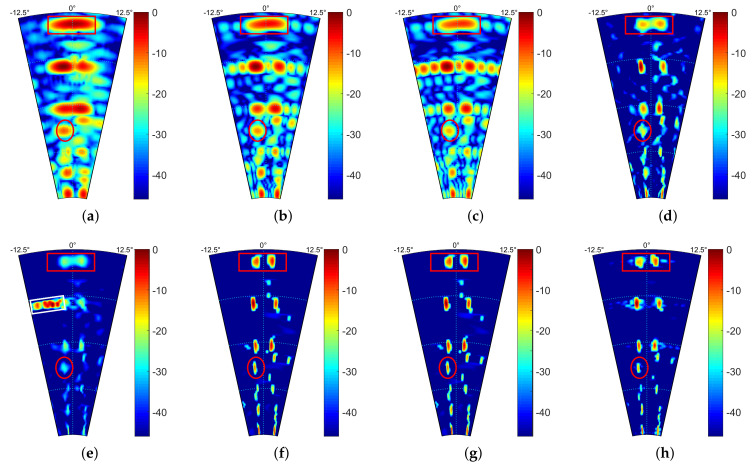
Processed results of Ginkgo Avenue data. (**a**) Real beam echo, (**b**) Result of TREGU, (**c**) Result of TSVD, (**d**) Result of RL, (**e**) Result of IAA, (**f**) Result of SBA, (**g**) Result of FSBA, (**h**) Result of SFSBA.

The experimental results are shown in Figure 7, where Figure 7a is the real beam echo with low resolution. We can find that many adjacent targets in the same range unit cannot be distinguished. For example, through prior information, we know that there are two trees in the area marked by the white rectangle, but we cannot distinguish them using real beam echo. Figure 7b–h show the processed results of different algorithms. It can be seen that the TREGU and TSVD only achieved limited resolution improvement, as Figure 7b,c shows. RL and IAA can distinguish most of the adjacent targets, but the targets in the white rectangle still can not be distinguished. Although it seems that the result of IAA is better than that of RL, there are false targets, as shown in the white rectangle marked in Figure 7e. In contrast, SBA, FSBA and the proposed SFSBA greatly improve the resolution of real beam echo, and all adjacent targets in the same range unit are also distinguished, as Figure 7f–h show. In addition, it can be seen that FSBA and SBA achieve the same results, but the performance of SFSBA is slightly worse than SBA and FSBA, especially in terms of the noise suppression ability. However, compared with TREGU, TSVD, RL and IAA, the super-resolution performance of the proposed SFSBA is still better. Having said that, in order to achieve the super-resolution effect shown in Figure 7f–h, SBA and FSBA perform 150 iterations, while the proposed SFSBA only performs 18 iterations. It can be found that the proposed SFSBA improves the convergence rate of SBA and FSBA by 8 times.

**Figure 8:**
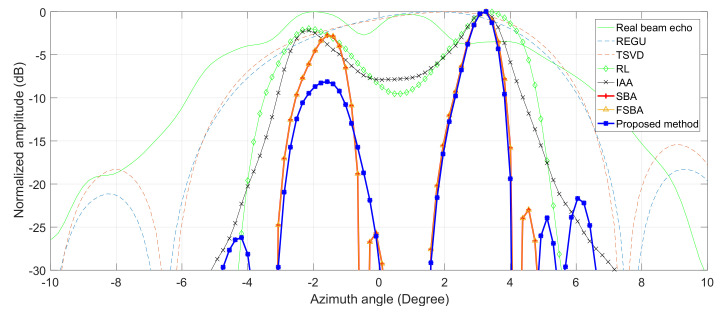
Profiles of local region in Figure 7.

The same as for the simulations, the profiles of the target marked in the white rectangle are shown in Figure 8. It can be seen that SBA, FSBA and the proposed SFSBA achieve higher resolution improvement when TREGU, TSVD, RL and IAA cannot distinguish adjacent targets. In addition, we also see that after acceleration, the noise suppression ability of the proposed SFSBA is worse than that of SBA and FSBA, but its performance is still better than TREGU, TSVD, RL, and IAA.

In order to quantitatively measure the resolution improvement, we select an isolated point target which is marked by a white circle to calculate the BSR of a different algorithm, and show it in Table 5. For the measured data, the BSR of SBA, FSBA and the proposed SFSBA is 7.1, which is much higher than other methods. Furthermore, the entropy of the super-resolution results of each algorithm is also listed in Table 5. It can be seen that the super-resolution results of SBA and FSBA are the same. The result of SFSBA is slightly worse than those of SBA and FSBA, but better than those of TREGU, TSVD, RL, and IAA.

#### 4.2.2. Real Data of Roof

**Figure 9:**
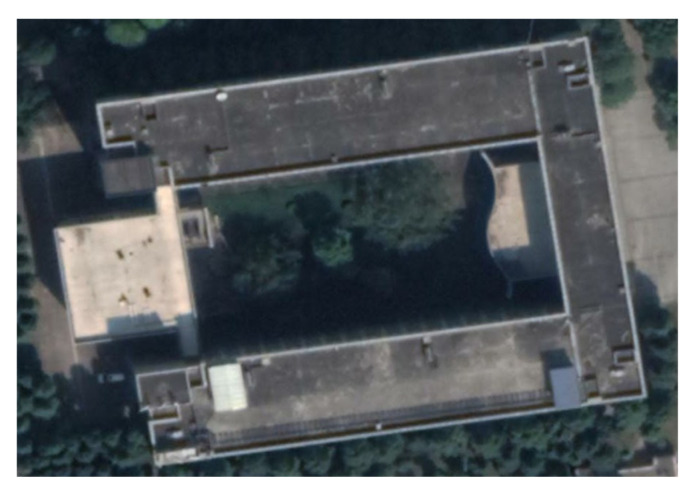
Optical scene of the roof.

Other real data were collected on a roof. The optical scene captured from Google Earth is shown in Figure 9. In this experiment, we placed some corner reflectors on the roof. The system parameters of this experiment are shown in Table 6.

**Figure 10:**
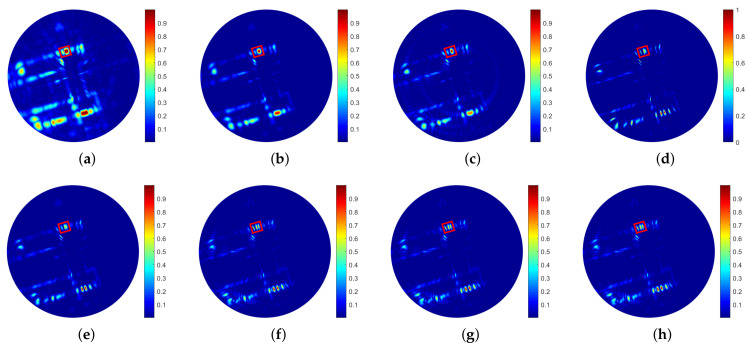
Processed results of roof data. (**a**) Real beam echo, (**b**) Result of TREGU, (**c**) Result of TSVD, (**d**) Result of RL, (**e**) Result of IAA, (**f**) Result of SBA, (**g**) Result of FSBA, (**h**) Result of SFSBA.

The experimental results are shown in Figure 10. Figure 10a is the real-beam echo with low SNR. From prior information, we know that the red rectangle marks three reflectors. However, they cannot be distinguished in the real-beam echo.

Figure 10b–e shows that TREGU, TSVD, RL and IAA can improve the resolution to a certain degree, but the reflectors marked by a red rectangle cannot be distinguished. From Figure 10f–h, it can be seen that SBA, FSBA and the proposed SFSBA achieve higher resolution improvement than other methods. The reflectors marked by red rectangle is distinguished clearly.

**Figure 11:**
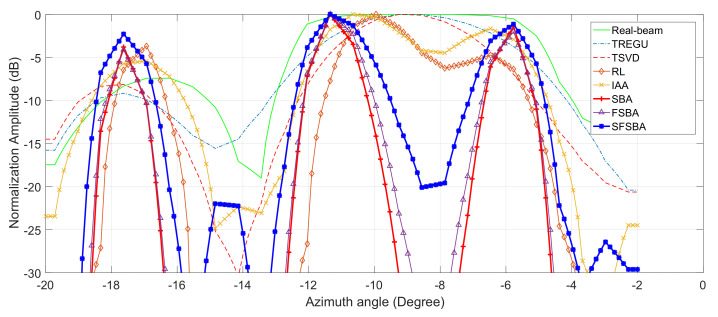
Profiles of local area in Figure 10.

The profiles of the area marked by a red rectangle are shown in Figure 11. It shows that only SBA, FSBA and the proposed SFSBA can clearly distinguish all the adjacent reflectors. Although the proposed SFSBA is slightly worse compared with SBA and FSBA, the noise level is lower than −20 dB. The super-resolution performance of the proposed SFSBA is much better than that of TREGU, TSVD, RL and IAA.

Similarly, the BSR and entropy of the real data are shown in Table 7. We can also see that the performance of SBA, FSBA and the proposed SFSBA is better than that of others, and the performance of SFSBA is similar to that of SBA and FSBA. It should be pointed out that the SNR of the roof data is higher than that of the Yinxin Avenue data (See Figure 7a and Figure 10a), so the processing effect of roof data is better.

### 4.3. Hardware Testing

Since the proposed SFSBA aims to improve the efficiency of the algorithm, in this subsection, we build a hardware platform based on a field programmable gate array (FPGA) to test its efficiency in practical application. The parameters of the FPGA are shown in Table 8.

For the simulation, the echo dimension is 292×400, where M×N denotes *M* range samples and *N* azimuth samples. For the real data, the dimensions are 120×35 and 220×500. Using the hardware platform for processing, the computing times (CTs) of different algorithms are shown in Table 9. The results show that the proposed SFSBA takes less time than other methods. The computational efficiency of the proposed SFSBA is 689, 103 and 712 times higher than that of the traditional SBA. Furthermore, the computational efficiency of SFSBA is about 8 times than that of FSBA, which will greatly improve the real-time super-resolution ability of radar in practical application.

## 5. Conclusions

Realizing radar forward-looking super-resolution imaging is extremely important in military and civil fields. Some traditional super-resolution methods face the problem of limited resolution improvement, such as TREGU, TSVD, RL and IAA. Although some methods can significantly improve the resolution, but suffer from high computational complexity or more iterations, such as SBA and FSBA, which cannot meet the real-time requirements in practice.

The SFSBA proposed in this paper overcomes these disadvantages. This method uses GS representation to reduce the computational complexity of each iteration, and the iterations are reduced by second-order vector extrapolation. Compared with TREGU, TSVD, RL and IAA, this method can not only effectively improve the azimuth resolution, but also has better computational efficiency than them. Compared with SBA, the computational complexity of each iteration is reduced from $O(N^{3})$ to $O(N^{2})$, and the number of iterations is also reduced by about 8 times. In addition, the computational efficiency of SFSBA is about 8 times that of FSBA. The super-resolution performance of SFSBA is only slightly worse than SBA and FSBA. In practical application, the proposed SFSBA can meet the requirements of resolution improvement and real-time performance.

Since the performance degradation of the SFSBA is mainly caused by vector extrapolation, in the future work, we will study higher-order vector extrapolation to minimize the performance degradation.

## Figures and Tables

**Table 1:** Flow chart of the proposed a superfast split Bregman algorithm (SFSBA).

Initialize: *k* = 0, $\sigma^{k}=y$, $g^{k}=0$, $b^{k}=0$
for *k*=1:2
$x^{k-1}=\mu H^{T}y+\lambda \left(g^{k-1}-b^{k-1}\right)$
$\sigma^{k}=WW^{H}x^{k-1}-TT^{H}x^{k-1}$
$g^{k}=\varsigma \left(\sigma^{k}+b^{k-1},1/\lambda \right)$
$b^{k}=b^{k-1}+\left(\sigma^{k}-g^{k}\right)$
end
for *k*=3:κ−1
$x^{k-1}=\mu H^{T}y+\lambda \left(g^{k-1}-b^{k-1}\right)$
$\sigma^{k}=WW^{H}x^{k-1}-TT^{H}x^{k-1}$
$d^{k-1}=\sigma^{k-1}-\sigma^{k-2}$
$d^{k-2}=\sigma^{k-2}-\sigma^{k-3}$
$\eta^{k}=\sqrt{\frac{{\left(d^{k-1}\right)}^{T}d^{k-1}}{{\left(d^{k-2}\right)}^{T}d^{k-2}}}$
$v^{k}=\sigma^{k}+\eta_{k}\nabla \sigma^{k}+\frac{1}{2!}\eta_{k}^{2}\nabla^{2}\sigma^{k}$
$g^{k}=\varsigma \left(v^{k}+b^{k-1},1/\lambda \right)$
$b^{k}=b^{k-1}+\left(v^{k}-g^{k}\right)$
end

**Table 2:** System parameters of simulation.

Parameter	Value	Units
Beam width	3.5	°
Band width	45	MHz
Antenna scanning velocity	50	°/*s*
Antenna scanning area	−5∼5	°
PRF	1000	Hz

**Table 3:** Performance index of simulation results.

Method	TREGU	TSVD	RL	IAA	SBA	FSBA	SFSBA
MSE ($\times 10^{-4}$)	71.67	46.34	9.01	15.44	4.34	4.34	4.37
BSR	2.45	3.33	7.36	14	25	25	17.5
Entropy	4.69	4.95	1.95	3.32	0.16	0.17	0.28

**Table 4:** System parameters of Ginkgo Avenue experiment.

Parameter	Value	Units
Beam width	5.1	°
Band width	75	MHz
Antenna scanning velocity	144	°/*s*
Antenna scanning area	−12.5∼12.5	°
PRF	200	Hz

**Table 5:** Performance indexes of real data processing.

Algorithm	TREGU	TSVD	RL	IAA	SBA	FSBA	SFSBA
BSR	1.21	2.08	1.86	2.09	7.1	7.1	7.1
Entropy	4.99	5.03	2.43	2.26	1.82	1.82	1.86

**Table 6:** System parameters of roof experiment.

Parameter	Value	Units
Beam width	5.1	°
Band width	45	MHz
Antenna scanning velocity	144	°/*s*
Antenna scanning area	0∼360	°
PRF	200	Hz

**Table 7:** Performance indexes of roof real data.

Algorithm	TREGU	TSVD	RL	IAA	SBA	FSBA	SFSBA
BSR	4.54	8.75	12.27	6.55	12.95	12.95	11.8
Entropy	3.34	3.30	3.82	3.71	2.62	2.62	2.93

**Table 8:** Parameters of the field programmable gate array (FPGA).

Parameters	Values
Chip	TMS320c6678
Manufacturer	Texas Instruments
Cores	8
Main frequency	1 GHz
Memory	4 GB

**Table 9:** Computing time of different algorithms.

Methods	TREGU	TSVD	RL	IAA	SBA	FSBA	SFSBA
CTs of simulation (s)	18.69	12.47	0.29	10.01	41.81	0.49	0.06
CTs of Ginkgo Avenue data (s)	0.059	0.039	0.09	0.097	0.311	0.25	0.003
CTs of roof data (s)	21.02	15.84	0.31	13.57	49.86	0.55	0.07

## Data Availability

Data sharing not applicable.
